# Practising person-centred care. Selected abstracts from the virtual 26th WONCA Europe conference, 6–10 July 2021

**DOI:** 10.1080/13814788.2021.1976752

**Published:** 2021-10-22

**Authors:** Jako S. Burgers, Bert Aertgeerts, Marjolein Y. Berger

**Affiliations:** a Dutch College of General Practitioners, Utrecht, The Netherlands; b Department of Family Medicine, Care and Public Health Research Institute (CAPHRI), Maastricht University, Maastricht, The Netherlands; c Department of Public Health and Primary Care, Academisch Centrum Huisartsgeneeskunde, KU Leuven, Belgium; d Department of General Practice & Elderly Care Medicine, University Medical Center Groningen, Groningen, The Netherlands

## Abstract

**Background:**

From 6 to 10 July 2021, WONCA Europe and the Dutch College of General Practitioners as host organiser welcomed 1,266 family physicians/general practitioners, teachers, researchers, and students from 66 countries interested in sharing knowledge, experience and innovations in primary healthcare.

**Methods:**

In cohesive sets of plenary presentations, round table sessions, and research masterclasses, aspects of patient care, research, and education around Practicing Person-Centred Care were presented and discussed. Actual topics in primary care such as covid-19, e-health and professional health, were covered in oral presentation sessions, one slide 5-minutes presentations, case presentations by young doctors and the e-poster gallery. All sessions were recorded and available on-demand for registrants until three months after the conference. All accepted abstracts have been published in the abstract book [https://www.woncaeurope.org/page/past-conference-abstract-books]. For this Journal, we selected the top 20 abstracts based on reviewers scores (mean of 3.5 or higher on a scale of 4) and consensus among members of the Scientific Committee.

**Results:**

The selected abstracts are divided into the following themes: (1) clinical topics often encountered in primary care, such as acute chest pain, urinary tract infections, dementia, and covid-19 (*N* = 5); (2) personalised care and related issues such as addressing multimorbidity (*N* = 2); shared decision making and patient empowerment (*N* = 4); (3) overdiagnosis and overtreatment, focusing on deprescribing (*N* = 2); (4) health promotion and prevention, including mental health (*N* = 2); (5) quality and safety (*N* = 2); (6) professional development and education (*N* = 1); (7) research and innovation, including teleconsultation (*N* = 2).

KEY MESSAGES

- The virtual 26th WONCA Europe conference 2021 was attended by 1,266 participants from 66 countries.
- Main theme was Practicing Person-Centred Care with special attention to actual topics such as covid-19 and e-health.
- All abstracts of the conference can be found at the WONCA Europe website https://www.woncaeurope.org/page/past-conference-abstract-books

## Introduction

In July 2021, WONCA Europe and the Dutch College of General Practitioners as host organiser welcomed 1,266 family physicians/general practitioners, teachers, researchers, and students from 66 countries interested in sharing knowledge, experience and innovations in primary healthcare. In cohesive sets of plenary presentations, round table sessions, and research master classes, aspects of patient care, research, and education around the theme Practicing Person Centred Care were presented and discussed. Other actual topics in primary care, such as covid-19, e-health, and professional health, were covered in oral presentation sessions, 1 slide 5 minutes presentations, case presentations by young doctors, and in the e-poster gallery. All sessions were recorded and were available on demand for registrants until three months after the conference.

All accepted abstracts are published in the abstract book *[https://www.woncaeurope.org/page/past-conference-abstract-books]*. For this Journal, we selected the top 20 abstracts based on the reviewers score (mean of 3.5 or higher on a scale of 4) and consensus among members of the Scientific Committee.

