# Supplementary material for: Practising person-centred care. Selected abstracts from the virtual 26th WONCA Europe conference, 6–10 July 2021
Source: Eur J Gen Pract. 2021 Oct 22;27(1):303–12. doi: 10.1080/13814788.2021.1976752 (PMC8682972; doi:10.1080/13814788.2021.1976752)
Supplement: Related texts

# Abstract: Sex differences in characteristics, triage assessment and clinical outcomes among patients with chest pain in urgent primary care

**Authors:** Amy Manten, Renee Bolijn, Remco Rietveld, Wim Lucassen, Irene Van Valkengoed, Henk Van Weert, Ralf Harskamp

**Affiliations:** a Department of General Practice, Amsterdam University Medical Centres, Amsterdam, Netherlands; b Department of Public and Occupational Health, Amsterdam University Medical Centres, Amsterdam, Netherlands; c Department of General Practice, Huisartsenorganisatie Noord-Kennemerland, Alkmaar, Netherlands

## Abstract

**Background:**

Telephone triage is fully integrated into urgent primary care in the Netherlands. The underlying triage protocols do not consider possible differences between men and women.

**Objectives:**

We aim to evaluate sex-specific differences for acute-onset chest pain, a key symptom in which adequate triage is pivotal.

**Methods:**

A retrospective cohort study of consecutive patients who contacted a regional, urgent primary care facility in Alkmaar, the Netherlands in 2017. We performed descriptive analyses on sex differences in patient and symptom characteristics, triage assessment and subsequent outcomes.

**Results:**

A total of 1,804 patients were included, the median age was 54 years and 57.5% were female. Women more frequently reported centrally located chest pain (32.2 vs. 27.7%), nausea (23.4 vs. 15.7%) and radiating pain to the back or jaw(s) (9.5 vs. 5.9% and 5.8 vs. 2.5%, respectively). Cardiovascular comorbidities were less common among women (47.5 vs. 54.3%). Triage urgencies were comparable between men and women, with comparable ambulance activation rates. However, women were more often visited at home (10.9 vs. 7.4%). At follow-up, women less often had an underlying cardiovascular condition (21.1 vs. 29.7%), including acute coronary syndrome (5.3 vs. 8.5%) compared to men.

**Conclusion:**

There are considerable differences between women and men who contact urgent primary care with chest pain. Notably, women have different symptom presentations, fewer cardiovascular risk factors, and lower risk of an underlying cardiovascular condition compared to men. Despite being at lower risk, ambulance activation is comparable between women and men.

# Abstract: Consultations and antibiotic treatment for urinary tract infections in Norwegian primary care 2006–2015, a registry-based study

**Authors:** Lars Emil Aga Haugom, Sabine Ruths, Knut Erik Emberland, Knut Eirik Ringheim Eliassen, Guri Rortveit, Knut-Arne Wensaas

**Affiliations:** a Research Unit For General Practice, NORCE Norwegian Research Centre AS, Bergen, Norway; b Department of Global Public Health and Primary Care, University of Bergen, Bergen, Norway

## Abstract

**Background:**

Extensive use of antibiotics and antimicrobial resistance is a major concern globally. In Norway, 82% of antibiotics are prescribed in primary care and one in four prescriptions are issued to treat urinary tract infections (UTI).

**Objectives:**

This study investigated time trends in antibiotic treatment following consultations for UTI in primary care.

**Methods:**

Registry-based study using linked data on all patient consultations for cystitis and pyelonephritis in general practice and out-of-hours (OOH) services and all dispensed prescriptions of antibiotics in Norway, 2006–2015.

**Results:**

Of the 2,426,643 UTI consultations, 94.5% were for cystitis and 5.5% for pyelonephritis; 79.4% were conducted in general practice and 20.6% in OOH services. From 2006 to 2015, annual numbers of cystitis and pyelonephritis consultations increased by 33.9 and 14.0%. Proportion of UTI consultations resulting in antibiotic prescription increased gradually for cystitis (36.6–65.7%) and pyelonephritis (35.3–50.7%). Cystitis was mainly treated with pivmecillinam (53.9%), and trimethoprim (20.8%) and pyelonephritis with pivmecillinam (43.0%), ciprofloxacin (20.5%) and sulfamethoxazole-trimethoprim (16.3%). For cystitis, the use of pivmecillinam increased the most (46.1–56.6%), and for pyelonephritis, the use of sulfamethoxazole-trimethoprim (11.4–25.5%) and ciprofloxacin (from 18.2–23.1%).

**Conclusion:**

During the 10-year study period, there was a considerable increase in UTI consultations resulting in antibiotic treatment. Treatment trend for pyelonephritis was characterised by more use of broader-spectrum antibiotics. These trends, indicative of enduring changes in consultation and treatment patterns for UTIs, will have implications for future antibiotic stewardship measures and policy.

# Abstract: Remote patient monitoring for COVID-19: impact on healthcare utilisation

**Authors:** Patricia Adam, Joshua Thompson, Derek Hersch

**Affiliations:** a Department of Family Medicine and Community Health, University of Minnesota Medical School, Minneapolis, MN, USA

## Abstract

**Background:**

In March 2020, MHealth Fairview System (12 hospitals and 60 clinics) deployed a remote patient monitoring (RPM) technology to increase access to care for patients with COVID-19 and reduce strain on in-person services. University of Minnesota Medical School faculty, residents and students served as the team monitoring patients and delivering care.

**Objectives:**

We studied the effect of this RPM solution on health care utilisation by patients with presumptive COVID-19.

**Methods:**

We conducted a retrospective study comparing health care utilisation by patients enrolled in the RPM program (*N* = 4,435) and those who declined enrollment (*N* = 2,742). Primary outcomes were ER visits, hospital and ICU admissions, and death. We used logistic regression to adjust for known risk factors of COVID-19 severity.

**Results:**

Adjusted for COVID-19 risk factors, there was a significant decrease in the risk of death for the group enrolled in the RPM: aOR:0.50 (95%CI:0.30,0.83). There were no significant associations between enrollment and the other primary outcomes. Increasing number of interactions with the RPM was associated with fewer hospital admissions: aOR:0.92 (95%CI:0.88,0.95).

**Conclusion:**

The COVID-19 pandemic strained health care systems and led to dramatic shifts in health care systems delivery in an attempt to alleviate this strain. The RPM was associated with reductions in hospitalisation, ICU admissions and most notably in death. More research is needed to determine if these technologies provide added benefits to traditional health care systems.

# Abstract: From COVID-19 respiratory clinic to vaccination hub: an adaptive model of primary healthcare

**Authors:** Karyn Elizabeth Alexander, Mukesh Chandra Haikerwal

**Affiliations:** a CIRQIT Health, T10, Altona North, Australia

## Abstract

**Background:**

Countries, facing the challenge of COVID-19, need to provide services that can adapt to fluctuations in demand and changing circumstances. In 2020, the Australian Government established 140 COVID-19 testing and screening ‘respiratory disease clinics’ across Australia. Our clinic, the first to open in Victoria, established a safe, effective and adaptive model of care employing a casual workforce of health assistants from multiple training backgrounds.

**Objectives:**

This paper describes a model of care that can adjust to continue to screen patients for COVID-19, whilst simultaneously rolling out vaccinations aimed at preventing infection.

**Methods:**

The respiratory clinic operates across two sessions, with three clinicians providing clinical services and Covid-19 testing. The patient transits through three phases of care after making an appointment:

 • Telephone registration and nurse-triage, from offices located above the clinic
 • In clinic assessment and testing, via drive-through or in-room consultation, determined clinically
 • Post-consultation notification of results to the patient and their regular GP, and data reporting.

Health assistants book appointments, guide patients, assist with infection control, remotely transcribe consultations (using video consultation from the clinic) and complete post-visit notifications. New staff are trained using a buddy system.

**Results:**

The adaptive model entails appointments for sessional vaccination clinics only when a nurse is satisfied that patients have enough information to provide informed consent. Patients attend nurse-led vaccinations in cohorts, guided and supported by health assistants. Vaccination details and notifications are gathered and transcribed remotely by video.

**Conclusion:**

This ‘Pandemic-flexible’ model of care, where health assistants support clinicians, can be further adapted to accommodate different clinical scenarios.

# Abstract: Hormone replacement therapy and dementia risk: Nested case-control studies using CPRD and QResearch

**Authors:** Yana Vinogradova, Tom Dening, Julia Hippisley-Cox, Lauren Taylor, Michael Moore, Carol Coupland

**Affiliations:** a Department of Primary Care, Medical School, University of Nottingham, Nottingham, United Kingdom; b Department of Psychiatry & Applied Psychology, Medical School, University of Nottingham, Nottingham, United Kingdom; c Nuffield Department of Primary Care Health Sciences, University of Oxford, Oxford, United Kingdom; d Department of Primary Care, Population Sciences and Medical Education, University of Southampton, Southampton, United Kingdom

## Abstract

**Background and purpose:**

Dementia is a progressive condition having major consequences for affected individuals, their families and carers. However, evidence on how hormone replacement therapy (HRT) – increasingly used by women affected by menopause – affects their risk of developing dementia is unclear. All previous studies have been relatively small short-term or have not accounted for some confounding variables.

**Methods:**

Two nested case-control studies used the UK primary care databases, QResearch and CPRD. Overall between 1998 and 2020, 118,501 women aged 55 and older were diagnosed with dementia and were matched by birth year and practice to up to five controls, alive and registered at the time of case diagnosis (index date). Exposure to HRT was based on prescriptions excluding those within three years prior to the index date. Risks for different types of HRT and duration of use were analysed using conditional logistic regression, adjusted for life-style factors, comorbidities and other drugs. Analyses were run for each database and results combined using meta-analysis techniques.

**Results:**

Overall, 16,291 (13.7%) cases and 68,726 (13.8%) controls were ever exposed to HRT. Overall, no associations were found between HRT use and dementia risk. However, a subgroup analysis of women diagnosed with Alzheimer’s disease demonstrated a small increased risk associated with oestrogen-progestogen therapy (odds ratio 1.08, 95% CI: 1.03–1.12), particularly for longer exposures (5–10 years: OR 1.11, 95% CI: 1.04–1.20; 10 years or more: OR 1.19, 95% CI: 1.06–1.33).

**Conclusion:**

This is the largest consistent study providing population-based risk estimates. The findings should assist doctors and patients considering HRT treatments.

# Abstract: Optima Forma – towards a patient-centred multimorbidity approach for chronic disease management in primary care

**Authors:** Lena Raaijmakers, Erik W. M. A. Bischoff, Jan Vercoulen, Tjard Schermer

**Affiliations:** a Primary and Community Care, Radboudumc, Nijmegen, Netherlands; b Medical Psychology, Radboudumc, Nijmegen, Netherlands

## Abstract

**Background:**

To reduce the burden of chronic diseases on society and individuals, European countries implemented disease management programmes (DMPs) that focus on a single chronic disease. However, (i) the scientific evidence that these DMPs reduce the burden of chronic disease in terms of health-related quality of life is not convincing, (ii) patients with multimorbidity may receive overlapping or conflicting treatment advice, and (iii) the single disease approach conflicts with the core competencies of primary care, i.e. medical generalistic, person-centred, and continuous care.

**Objectives:**

This study aimed to develop a holistic, person-centred and integrated approach for the management of patients with chronic diseases and multimorbidity in primary care.

**Methods:**

A mixed-methods study was conducted in the Netherlands from January 2019 to December 2020. First, we performed a scoping review to construct a theoretical model. Second, 57 healthcare professionals commented on the model in online qualitative questionnaires. Third, 9 patients with chronic conditions were interviewed on the model by phone. Finally, the model was presented to 3 local primary care cooperatives and finalised after their comments.

**Results:**

A stepwise software-supported approach was developed, including (i) assessing patient’s integral health status using (web-based) questionnaires and physical measurements; (ii) discussing the results with a case manager, after which (iii) treatment goals are formulated, suitable interventions in the primary care network are selected and an evaluation is planned.

**Conclusion:**

We developed a holistic, person-centred, integrated approach for managing patients with chronic diseases and multimorbidity in primary care. This approach will be tested in a pilot study in 2021 to establish its feasibility and potential effects.

# Abstract: Person-centred care in primary care: What works for whom, how, and in what circumstances?

**Authors:** Anam Ahmed, Maria Van Den Muijsenbergh, Bert Vrijhoef

**Affiliations:** a Panaxea, Amsterdam, Netherlands; b Primary and Community Care, Radboud University Medical Centre, Nijmegen, Netherlands; c Prevention and Care, Pharos, Utrecht, Netherlands; d Patient and Care, Maastricht University Medical Centre, Maastricht, Netherlands

## Abstract

**Background:**

Person-centred care (PCC) is the cornerstone of primary care. However, insights into when PCC does (not) work, for whom, why and how, are lacking.

**Objectives:**

This study aimed to identify the relationships between the context, the mechanisms, and the outcomes resulting from this interaction using rapid realist review (RRR).

**Methods:**

Peer-reviewed and non-peer-reviewed literature reporting on PCC in primary care were included. Selection and appraisal of documents was based on relevance and rigour according to the Realist and Meta-Review Evidence Synthesis: Evolving Standards (RAMESES) guidelines. Data on context, mechanisms, and outcomes (CMO) were extracted. CMO-configurations were set up to establish a programme theory (PT).

**Results:**

The PT demonstrates interaction of multiple context items, such as addressing PCC (including (low) health literacy) in the care policy, patients having a social support network, and training of healthcare providers (HCPs). Information technology optimisation is needed to tailor patient information, make it available, and to integrate information for HCPs. Mechanisms include taking into account the patient preferences and social/cultural differences, involving patient groups in the organisation of care and developing (new) tools, building solid collaborations between HCPs and patients to stimulate shared decision-making, and offering tailored communication and self-management support. The interaction between context and mechanisms lead to higher compliance, better health outcomes, and provision of tailored care.

**Conclusion:**

Embedding all factors within their mutual coherence is necessary for effective PCC in primary care. In doing so, this study proposes a PT.

# Abstract: Developing a tool for patient involvement in general practice: The preparing patients for active involvement in medication review (PREPAIR) tool

**Authors:** Amanda Sandbæk, Marlene Christina Rosengaard Møller, Flemming Bro, Kirsten Høj, Line Due Christensen, Anna Mygind

**Affiliations:** a Department of Public Health, Aarhus University, Aarhus C, Denmark; b The Research Unit for General Practice, Aarhus C, Denmark

## Abstract

**Background:**

Active patient involvement can contribute to improved treatment outcomes and more patient-centred care. Yet, patient involvement remains a challenge in clinical practice.

**Objectives:**

We aimed to develop a new tool, the PREparing Patients for Active Involvement in medication Review (PREPAIR) tool, to enhance systematic patient involvement in conversations about medication optimization in general practice.

**Methods:**

A literature review was conducted and followed by co-producing activities: (1) a workshop with six GPs and (2) pilot testing including observations and interviews with 22 patients, three GPs and three staff members. During this process, continuous adaptations of the PREPAIR were made.

**Results:**

The final tool included five questions: (1) satisfaction with current medications, (2) experience of taking too much medication, (3) major side effects, (4) experience of taking unnecessary medication, and (5) medication-related topics to discuss with the GP (open-ended question). The PREPAIR tool was completed by the patient before the GP consultation to encourage patient reflections on own medications. During the consultation, the GP’s focus changed from the computer towards the patient, questionnaire responses were reviewed, and potential medication-related problems were discussed. The patients were empowered to speak, and the GPs improved their understanding of patient perspectives on medications. Although some GPs suggested a broader scope on health perspectives, the PREPAIR tool was received positively by both patients and GPs.

**Conclusion:**

We developed a brief and valuable tool to support systematic patient involvement in general practice. Future research should address whether the PREPAIR tool can contribute to improved patient outcomes and quality of care.

# Abstract: A systematic approach to identify and prioritise option tables for recommendations in NHG-guidelines

**Authors:** Anouk Baghus, Gerda Van Der Weele

**Affiliations:** a Department of Family Medicine, Maastricht University, Maastricht, Netherlands; b Guideline Development, Dutch College of General Practitioners, Utrecht, Netherlands

## Abstract

**Background:**

The Dutch College of General Practitioners (NHG) has a longstanding comprehensive guideline programme, including 135 clinical practice guidelines. In recent years, these guidelines are adapted to facilitate shared decision making, including the integration of decision support tools, such as option tables.

**Objectives:**

To guide the development of option tables, we identified preference-sensitive recommendations within the clinical practice guidelines, for which option tables are most needed, as resources are limited.

**Methods:**

We systematically analysed all 135 NHGs clinical practice guidelines and selected all preference-sensitive recommendations. Based on several criteria, we then assessed for which recommendations an option table can have added value. Recommendations for which an option table is not yet available or under development were added to the longlist. In the upcoming months, this longlist will undergo prioritisation by several stakeholders such as general practitioners and patient(organisation)s.

**Results:**

We identified 87 preference-sensitive recommendations for which an option table can have added value according to our criteria. For 10 recommendations, an option table is already available or under development in primary or multidisciplinary care. The remaining 77 recommendations will undergo prioritisation. The results of this prioritisation were presented at WONCA, July 2021.

**Conclusion:**

We systematically identified and prioritized preference-sensitive recommendations within NHGs clinical practice guidelines, for which an option table can have added value. This will guide us to make sensible choices for a programmatic approach of the development of option tables.

# Abstract: Dutch general practitioners’ views on an (early) dementia diagnostic trajectory and implementation of shared decision making therein: A qualitative interview study

**Authors:** Iris Linde, Claire Wolfs, Marieke Perry, Maud Hevink, Job Metsemakers, Trudy van der Weijden, Marjolein de Vugt, Frans Verhey, Ron Handels, Marcel Olde Rikkert, Carmen Dirksen, Rudolf Ponds

**Affiliations:** a Department of Psychiatry and Neuropsychology, School For Mental Health and Neuroscience (MHENS), Maastricht University/Alzheimer Centre Limburg, Maastricht, Netherlands; b Department of Geriatric Medicine, Radboudumc, Nijmegen, Netherlands; c Department of Family Medicine, Maastricht University, Maastricht, Netherlands; d Department of Geriatric Medicine, Donders Institute For Brain Cognition and Behaviour, Radboud University Medical Center, Nijmegen, Netherlands; e Clinical Epidemiology and Medical Technology Assessment (KEMTA), Maastricht University Medical Centre, Maastricht, Netherlands; f Department of Medical Psychology, Maastricht University Medical Centre, Maastricht, Netherlands

## Abstract

**Background:**

Clinicians, researchers, and Alzheimer Societies stress the importance of an early dementia diagnosis in a mild stage. However, whether this is an improvement in patients’ health and well-being is still debated. Ideally, shared-decision making (SDM) is implemented to discuss the potential benefits and harms of an early diagnosis and disclosure with patients.

**Objectives:**

This study explores experiences and considerations of GPs regarding an (early) diagnostic trajectory for dementia and implementation of SDM therein.

**Methods:**

In this qualitative study, GPs and practice-based nurses were interviewed. Topics included views concerning early dementia diagnosis, the decision-making process for starting a diagnostic trajectory, and views on the implementation of SDM in this regard.

**Results:**

Sixteen GPs and practice-based nurses in the Netherlands were interviewed. Several considerations concerning the timing of a dementia diagnosis were identified, including; (1) decrease in patients’ quality of life (QoL) due to an (early) diagnosis, (2) potential advantages of an early diagnosis for patients and their significant others (3) the possibility of a misdiagnosis (4) experiences related to a dementia diagnosis in a late disease stage.

Several patient and dyad related factors were identified that could hinder or facilitate the implementation of SDM in general practice.

**Conclusion:**

Most GPs favoured a timely (instead of an early) diagnostic trajectory (i.e. initiated at the right time for patients and significant others to meet their needs and expectations) and emphasised the importance of their patients’ QoL. GPs favoured patient involvement in deciding on an (early) diagnostic trajectory, but several barriers and facilitators (e.g. patients’ expectations regarding treatment) affect SDM.

# Abstract: GPinfo.nl: Supporting international patients and their general practitioners in the Netherlands with independent and reliable digital health information in English

**Authors:** Freke Zuure, Patrick Jansen, Jantine Woudstra, Marjolein Van Lennep, Mariella Schreur, Johannes De Boer, Lidy Pols, Dirk Jan Frijling

**Affiliations:** a Practice, Quality and Innovations, Dutch College of General Practitioners (NHG), Utrecht, Netherlands; b Department of General Practice, SGE International, Eindhoven, Netherlands; c Cooperative General Practitioners Amsterdam Groot-Zuid, Chagz, Amsterdam, Netherlands; d Healthcare for Internationals, H4i, Eindhoven, Netherlands

## Abstract

**Background:**

Foreign-born persons unfamiliar with Dutch health practices have less confidence in the Dutch healthcare system and the GP. Cultural and language barriers can impede good delivery of care. Thuisarts.nl, the patient information website of the Dutch College of General Practitioners, offers reliable and easy-accessible health information. Thuisarts.nl supports GPs with patient education and improves patient empowerment.

**Objectives:**

We evaluated the usage and perceived usefulness of an English pilot-version (GPinfo.nl) among internationals (i.e. foreign-born persons living in the Netherlands for work or study) and GPs in the Netherlands.

**Methods:**

GPinfo.nl offers a selection of medical (*n* = 19) and Dutch-healthcare topics (*n* = 15), 1:1 translated from Thuisarts.nl. Newsletters and the Healthcare for internationals (H4i.nl) network promoted GPinfo.nl among GPs. A limited-budget social-media and Google-Adwords campaign was set up to reach internationals. The website was monitored from September-December 2020 using web-statistics, an online exit-survey (*N* = 148), interviews (*N* = 6) among internationals, an online survey (*N* = 68) and interviews (*N* = 6) among GPs.

**Results:**

GPinfo.nl had 20,342 visits. 79% (95%CI =72-85%) and 78% (95%CI =72-85%) of internationals perceived GPinfo.nl to be reliable and relevant, respectively. 70% (95%CI =59-81%) and 77% (95%CI =67-87%) of GPs indicated that GPinfo.nl improved their interaction with international patients and the efficiency of their consultations, respectively. Internationals and GPs encouraged the initiative. Their suggested improvements for more impact were: more healthcare-system-related information and explanation of Dutch medical practices, greater coverage of medical subjects and incorporation of cross-cultural sensitivity.

**Conclusion:**

This pilot confirmed the need for and potential impact of health(care) information in English for internationals and GPs. However, a 1:1 translation of Thuisarts.nl seems yet insufficient and adaptation is required.

# Abstract: Deprescribing in the elderly, a multidisciplinary guideline

**Authors:** Amanda S. G. Van Walraven, Monique M. Verduijn

**Affiliations:** a Guideline Development, Dutch College of General Practitioners, Utrecht, Netherlands

## Abstract

**Background:**

Medical guidelines often describe the initiation of chronic medication but usually not the process of discontinuation in ageing patients. Like prescribing medication, reducing or stopping medication should be part of daily medical practice, especially in vulnerable elderly patients.

**Objectives:**

The aim was to develop a generic guideline on deprescribing in the elderly, supported by 10 factsheets on deprescribing frequently used medicines in the elderly.

**Methods:**

The guideline has been developed by a multidisciplinary working group. The development process was based on the AGREE-II model. Since there is still little research from the perspective of patients and care providers on deprescribing, a mixed-method was followed with subsequent steps:

 • literature review
 • focus groups: patients and caregivers
 • focus groups: health care providers
 • narrative summary of publications on ethical and disciplinary aspects of reduction and stopping medication

**Results:**

A multidisciplinary guideline was developed with recommendations on:

 • the impeding and facilitating factors for deprescribing;
 • the effects of deprescribing;
 • instruments for deprescribing;
 • suitable patients and suitable moments
 • division of roles among healthcare providers in deprescribing In addition, 10 fact sheets have been developed detailing the above points about deprescribing common medicines in the elderly.

**Conclusion:**

The goal of reducing and stopping medication is to optimise drug treatment and thus improve the quality of life and health of the patient. The development of the guideline ‘deprescribing in the elderly’ and 10 corresponding factsheets offer GPs, other prescribers, pharmacists and patients tools for reducing and discontinuing medicines in elderly patients (≥70 years) in daily practice.

# Abstract: ‘Never change a winning team:’ GPs’ perspectives on discontinuation of long-term antidepressants

**Authors:** Ellen Van Leeuwen, Sibyl Anthierens, Mieke Van Driel, An De Sutter, Evelien Van Den Branden, Thierry Christiaens

**Affiliations:** a Department of Basic and Applied Medical Sciences + Department of Public Health and Primary Care, Ghent University, Ghent, Belgium; b Department of Family Medicine and Population Health, University of Antwerp, Antwerpen, Belgium; c Faculty of Medicine, Primary Care Clinical Unit, The University of Queensland, Brisbane, Australia; d Department of Public Health and Primary Care, Ghent University, Ghent, Belgium; e General Practice Het Spoor, Het Spoor, Aalst, Belgium; f Department of Basic and Applied Medical Sciences, Ghent University, Ghent, Belgium

## Abstract

**Background:**

Long-term antidepressant use, much longer than recommended by guidelines, can harm patients and generate unnecessary costs. Most antidepressants are prescribed by general practitioners (GPs) but it remains unclear why they do not discontinue long-term use.

**Objectives:**

This study aims to explore GPs’ views and experiences of discontinuing long-term antidepressants, barriers and facilitators of discontinuation and required support.

**Methods:**

This is a qualitative study including 20 semi-structured face to face interviews with Belgian GPs. Interviews were analysed thematically.

**Results:**

The first theme, ‘Success stories’ describes three strong motivators to discontinue antidepressants: patient health issues, patient requests and a new positive life event. Second, not all GPs consider long-term antidepressant use a ‘problem’ as they perceive antidepressants as effective and safe. GPs’ main concern is risk of relapse. Third, GPs foresee that discontinuation of antidepressants is not an easy and straightforward process. GPs weigh up whether they have the necessary skills and whether it is worth the effort to start this process.

**Conclusion:**

Discontinuation of long-term antidepressants is a complex and uncertain process for GPs, especially in the absence a facilitating life-event or patient demand. Absence of a compelling need for discontinuation and fear of relapse of symptoms in a stable patient are important barriers for GPs when considering discontinuation. To increase GPs’ motivation to discontinue long-term antidepressants, more emphasis on the futility of the actual effect and potential harms related to long-term use is needed.

# Abstract: How do general practitioners handle couple relationship problems? A focus-group study from Norway

**Authors:** Siri Dalsmo Berge, Mette Brekke, Eivind Meland, Thomas Mildestvedt

**Affiliations:** a Department of Global Public Health and Primary Care, University of Bergen, Bergen, Norway; b Department of General Practice, University of Oslo, Oslo, Norway

## Abstract

**Background:**

Couple relationship satisfaction is related to physical and mental health and longevity. In a Norwegian study from 2020, one in four patients reported talking about their couple relationship with their general practitioner (GP).

**Objectives:**

This study explores how GPs’ experience couple relationship problems and how they identify patients with couple relationship problems.

**Methods:**

This is an exploratory qualitative study. In 2020, we conducted three semi-structured focus group interviews with 18 GPs. We developed a semi-structured interview guide and used systematic text condensation for the inductive analyses.

**Results:**

All participating GPs reported an abundance of experiences handling couple relationship problems in their practice. These issues both served as explanation for relevant clinical problems and were important in a holistic approach to the patients and their families. The GPs had different amounts of training in psychotherapy. Some emphasised that doctors learn communication skills useful for individual consultations in medical education but do not develop skills for dyadic counselling. The most experienced GPs felt qualified to support patients with couple relationship problems, though none of the participants could see themselves as couples therapists. Some wanted specific tools for the GP to use when this issue is brought up.

**Conclusion:**

Experienced GPs are comfortable having supportive conversations with individual patients regarding couple relationship problems. GPs could need more skills in coping with patients’ relational problems and dyadic counselling. A first-aid-kit for GPs facing patients with couple relationship problems might be useful.

# Abstract: Perception of primary health care response capacity by patients suffering mental health problems and without them: A qualitative study

**Authors:** Fátima Méndez, Bárbara Oliván-Blázquez, Antonia Sánchez-Calavera, Josefa Gil-De-Gómez, Eva Rodríguez-Eguizabal, Valle Coronado-Vázquez, Rosa Magallón-Botaya

**Affiliations:** a Aragonese Research Group In Primary Care (GAIAP), Institute for Health Research Aragón (IIS Aragón), Zaragoza, Spain; b Department of Psychology and Sociology, Faculty of Social and Labour Sciences, University of Zaragoza, Zaragoza, Spain; c Primary Care Center, Aragon Health Service, Zaragoza, Spain; d Primary Care Center, Rioja Health Service, La Rioja, Spain

## Abstract

**Background:**

Health Systems’ Response Capacity (HSRC) is defined as the ‘ability of the health system to meet the population’s legitimate expectations regarding their interaction with the health system, apart from expectations for improvements in health or wealth.’ HSRC is measured through eight domains: dignity, confidentiality, communication, autonomy of individuals, prompt attention, basic quality of facilities, access to social support networks, and choice of care providers.

**Objectives:**

This study aims to deepen Primary Health Care Response Capacity by specifically using patients suffering from a mental disorder, and without these health problems.

**Methods:**

Qualitative methodology. For this study, in-depth interviews were conducted with 28 patients with and without mental health disorders. An inductive thematic content analysis by pairs was performed using grounded theory to explore, develop and define the analysis.

**Results:**

The fundamental domains for patients are dignity, communication, and rapid attention. People with mental health problems also highlight the domain of confidentiality as relevant while patients who do not have a mental health problem prioritise the domain of autonomy. Patients with mental health disorders report a greater number of negative experiences about the domain of dignity. The interrelationship between domains also appears in the discourses, with a relationship between clear communication, autonomy, dignity.

**Conclusion:**

The prevalence of patients with mental illness who use primary care is quite high; therefore, it is necessary to determine the factors that influence its responsiveness to plan the resources to be offered to this population at this early care level.

# Abstract: Which practice characteristics are associated with personal continuity?

**Authors:** Marije T. te Winkel, Pauline Slottje, Birgit I. Lissenberg-Witte, Rob J. vanMarum, Henk J. Schers, Annemarie A. Uijen, Jettie Bont, Otto R Maarsingh

**Affiliations:** a Department of General Practice, Amsterdam UMC, Amsterdam, Netherlands; b Department of Epidemiology and Biostatistics, Amsterdam UMC, Amsterdam, Netherlands; c Medicine For Older People, Amsterdam UMC, Amsterdam, Netherlands; d Primary and Community Care, Radboud University Nijmegen Medical Centre, Nijmegen, Netherlands

## Abstract

**Background:**

Continuity of care has many benefits, including a better patient-doctor relationship, prevention of hospital admission, reduced health care costs, better medication compliance, and lower mortality rates.

**Objectives:**

This study investigates the association between personal continuity and general practice characteristics and identifies additional factors associated with personal continuity.

**Methods:**

Observational study of 4.7 million contacts of 190.886 patients from 48 different general practices in 2013–2018 in the Netherlands. Personal continuity was calculated using four established measures (Usual Provider Continuity; Bice-Boxerman Continuity of Care Index; Herfindahl Index; Modified Modified Continuity Index). Linear mixed models were used to determine the association between continuity level and practice characteristics, adjusted for patient characteristics. To identify additional factors associated with personal continuity, we conducted interviews with general practitioners working in practices with the largest difference between observed and predicted continuity.

**Results:**

We identified nine practice characteristics significantly associated with continuity of care (*p* < 0.05). Of these characteristics, six were significant in all models: number of registered patients; number of doctors and their working days; number of locums and their percentage of contacts; number of other employees. These effects were adjusted for patient characteristics, including type of contacts, age, sex, medical history, time of registration, and ancestry (*p* < 0.05). Interviews may provide insights in additional factors (analyses will be completed).

**Conclusion:**

Six characteristics were associated with higher continuity of care, which can be calculated using general practice data. Optimisation of these characteristics may contribute to improving personal continuity.

# Abstract: Patient complaints as a source for improving patient safety in primary care facilities

**Authors:** Maria-Pilar Astier-Peña, Ana MaríaMartínez Gilabert, Carlos Clerencia-Casorran, Maria Elena Lain-Miranda, Gloria DianaSerrano Peris, José Miguel Bueno-Ortiz

**Affiliations:** a Professionalism and Clinical Ethics, University of Zaragoza, Zaragoza, Spain; b Central Support Services, Aragon Health Care Service, Zaragoza, Spain; c Primary Care Directorate. Sector Zaragoza I, Aragon Health Care Service, Zaragoza, Spain; d Primary Care Directorate. Sector Zaragoza Iii, Aragon Health Care Service, Zaragoza, Spain; e Fuente Alamo Health Center, Murcia Health Care Service, Alcantarilla, Spain

## Abstract

**Background:**

Primary care professionals (PCP) in Spain have a meager rate of reporting patient safety incidents (PSI). Care pressure and reporting burden are disincentives. Patients’ complaints at primary care facilities regarding their care can be used as a source of PSI.

**Objectives:**

This study aims to analyse patient complaints (PC) in 4 health areas of Aragon Health Care Service (Spain) – covering 958,000 inhabitants- and identify those related to patient safety issues.

**Methods:**

A simple random sampling was performed regarding all the claims issued in 2017 for primary care facilities. The total sample was 324 claims. Peer review was made with a checklist to identify PSI considering: sex and age of the patient, nature of PSI, professional involved, place of healthcare, severity, and avoidability of harm. Claims were reviewed by two researchers (kappa concordance test 0.94) and a third independent reviewer for disagreements. The three were family doctors.

**Results:**

24.3% (84/324) of claims are due to PSI. Family Doctors received 44.9% of claims. Among the PSI detected (84), 52.3% were due to problems in the healthcare process and 23.5% to coordination between care levels and waiting times. Regarding causal factors, 51% involved diagnosis and 24.9% management and communication issues. A total of 39/84 were PSI with harm, of whom 4/84 (6%) were moderate and 2/84 serious harm. A total of 2.6% (1/39 PSI) were unavoidable while 97.4% (38/39 complaints with PSI) could have been avoided.

**Conclusion:**

Patients’ claims content PSI. The analysis and follow-up of PSI claims can be considered as a source of information to improve PS in primary care.

# Abstract: Developing educational resources to improve primary healthcare services for people with deafness and hearing loss

**Authors:** Devina Maru Royal

**Affiliations:** a College of General Practitioners, National Clinical Champion For Deafness and Hearing Loss, London, United Kingdom

## Abstract

**Background:**

Twelve million people have hearing loss in the UK. GPs have identified a lack of training in the necessary skills to communicate effectively with people who have hearing loss, and this has presented a clear learning gap. Evidence suggests that people wait on average 10 years before seeking help for hearing loss and 30-45% presenting to their GP are not referred to NHS audiology services.

**Methods:**

To address this knowledge gap, a diverse stakeholder group was formed with The Royal National Institute for Deaf People, NHS England/Improvement, RCGP and the patient public voice community to tackle the barriers impacting hearing health and to raise awareness. A toolkit of educational resources was developed, including podcasts, videos, screencasts, online courses, which GPs, trainees and members of the wider primary care team could access, engage and receive appropriate training and support.

**Results:**

19,850 CPD tinnitus module users; 3,659 podcast listens; 2,576 number of toolkit views. RCGP core curriculum updated with references to hearing loss. RCGP accredited its first Deaf awareness online course for doctors. NHS England bulletin and BJGP article published a piece highlighting the educational resources for use in primary care.

**Conclusion:**

Resources developed give GPs and trainees the confidence to recognise the symptoms of hearing loss and appropriately refer for a hearing assessment promptly. It signposts resources to help with remote consulting and covers new additions of the core curriculum to hearing loss. The toolkit provides QI initiatives and helps GP surgeries comply with legislation (Accessible Information Standard and Equality Act), which CQC inspects.

# Abstract: Teleconsultation, a tool for the future? – the Portuguese family doctors’ perspective

**Authors:** Andreia Cunha Castro, Nelson Campos, Susana DeSá Laranjeira, Cristina Ramos Silva, Elodie Santos, Rosana Dantas, José Pedro Águeda

**Affiliations:** a ACeS Grande Porto IV (Póvoa de Varzim/Vila do Conde), Usf Do Mar, Póvoa de Varzim, Portugal; b ACeS Cávado I (Braga), Usf + Carandá, Braga, Portugal; c ACeS Grande Porto IV (Póvoa de Varzim/Vila do Conde), Usf Casa Dos Pescadores, Póvoa de Varzim, Portugal; d ACeS Grande Porto IV (Póvoa de Varzim/Vila do Conde), Usf Das Ondas, Póvoa Varzim, Portugal; e ACeS Cávado I (Braga), Usf Ruães, Mire de Tibães, Portugal; f ACeS Baixo Mondego, Ucsp Mira, Mira, Portugal

## Abstract

**Background:**

In the context of the 2020 SARS-CoV-2 pandemic, healthcare services had to get reorganised and resorted to teleconsultation in their clinical practice.

**Objectives:**

This study’s primary goal is to know the Portuguese Family Doctors’ perspective on the utility of teleconsultation in their future clinical practice. Second, to recognize advantages and disadvantages of teleconsultation, identify possible difficulties and under what circumstances it may represent added value.

**Methods:**

A cross-sectional study was realised between September 2020 and January 2021 through the application of an online questionnaire. The target population was family medicine physicians and residents who worked in primary healthcare belonging to the Portuguese National Health Service.

**Results:**

Overall, 377 responses were received of which 83.6% considered that teleconsultation represented a valuable asset to the future of medicine. The main identified advantage was the greater accessibility by patients to healthcare (68.7%) and the main disadvantage was the impossibility to perform a proper physical examination (81.2%). The lack of appropriate resources, like phone line, camera, internet connection and suitable software was considered a primary barrier (70%). A total of 47.8% participants answered that the relative time needed for a teleconsultation should be the same as a usual consultation of the same typology. The type of consultation considered the most appropriate to telemedicine was Adult Health (68.7%).

**Conclusion:**

In the Portuguese family physicians’ perspective, teleconsultation will become essential to the future of primary care even though it will take some time to understand its best applicability.

# Abstract: Assessment of the use of retinography as a screening method for the early diagnosis of chronic glaucoma in primary care

**Authors:** Soraya Sánchez González

**Affiliations:** a Servicio Andaluz de Salud, Suap- Bollullos Del Condado (Huelva), Huelva, Spain

## Abstract

**Background:**

Glaucoma is an optic neuropathy characterised by morphological ocular changes and alterations in visual functions. Rapid diagnosis is necessary to initiate treatment to halt the advance of glaucoma towards blindness.

**Objectives:**

This PhD study aims to determine usefulness, validity of retinographies performed in primary care as a tool for early diagnosis of open-angle chronic glaucoma (OAG).

**Methods:**

An observational, descriptive and cross-sectional study with two blinded parallel observers (2 general practitioners and 1 ophthalmologist), developed in an urban Primary Care Health Centre and the Ophthalmology Department outpatient clinic. A total of 196 patients of both genders, between 40 and 70 years, with diabetes and hypertension, and undiagnosed with glaucoma, were recruited by phone call. Two patients that did not arrive for their appointments for the ophthalmology tests were considered as losses.

**Results:**

The retinography for OAG screening has a sensitivity of 21% (95% CI: 0–43%), a specificity of 93% (95% CI: 89–97%), a negative predictive value of 94% (95% CI: 90–97%), and positive of 20% (95% CI: 0–40%); positive probability ratio of 3.07 (95% CI: 0.98–9.62) and negative 0.84 (95% CI: 0.64–1.11). The IC was 0.653 (95% CI: 0.495–0.769) and kappa index of 0.140 (0.106 ET).

**Conclusion:**

According to this proposed model, retinography is not a valuable tool for the early diagnosis of OAG in primary care, as it is not safe enough. Before it can be used, it would need adjustments for its low sensitivity and use of other combined tests. The training of general practitioners would also need to be improved.
